# A metabolomics comparison of plant-based meat and grass-fed meat indicates large nutritional differences despite comparable Nutrition Facts panels

**DOI:** 10.1038/s41598-021-93100-3

**Published:** 2021-07-05

**Authors:** Stephan van Vliet, James R. Bain, Michael J. Muehlbauer, Frederick D. Provenza, Scott L. Kronberg, Carl F. Pieper, Kim M. Huffman

**Affiliations:** 1 Duke Molecular Physiology Institute, Duke University Medical Center, Durham, NC USA; 2 Department of Wildland Resources, Utah State University, Logan, UT USA; 3 Northern Great Plains Research Laboratory, USDA-Agricultural Research Service, Mandan, ND USA

**Keywords:** Metabolism, Physiology, Medical research

## Abstract

A new generation of plant-based meat alternatives—formulated to mimic the taste and nutritional composition of red meat—have attracted considerable consumer interest, research attention, and media coverage. This has raised questions of whether plant-based meat alternatives represent proper nutritional replacements to animal meat. The goal of our study was to use untargeted metabolomics to provide an in-depth comparison of the metabolite profiles a popular plant-based meat alternative (n = 18) and grass-fed ground beef (n = 18) matched for serving size (113 g) and fat content (14 g). Despite apparent similarities based on Nutrition Facts panels, our metabolomics analysis found that metabolite abundances between the plant-based meat alternative and grass-fed ground beef differed by 90% (171 out of 190 profiled metabolites; false discovery rate adjusted p < 0.05). Several metabolites were found either exclusively (22 metabolites) or in greater quantities in beef (51 metabolites) (all, p < 0.05). Nutrients such as docosahexaenoic acid (ω-3), niacinamide (vitamin B3), glucosamine, hydroxyproline and the anti-oxidants allantoin, anserine, cysteamine, spermine, and squalene were amongst those only found in beef. Several other metabolites were found exclusively (31 metabolites) or in greater quantities (67 metabolites) in the plant-based meat alternative (all, p < 0.05). Ascorbate (vitamin C), phytosterols, and several phenolic anti-oxidants such as loganin, sulfurol, syringic acid, tyrosol, and vanillic acid were amongst those only found in the plant-based meat alternative. Large differences in metabolites within various nutrient classes (e.g., amino acids, dipeptides, vitamins, phenols, tocopherols, and fatty acids) with physiological, anti-inflammatory, and/or immunomodulatory roles indicate that these products should not be viewed as truly nutritionally interchangeable, but could be viewed as complementary in terms of provided nutrients. The new information we provide is important for making informed decisions by consumers and health professionals. It cannot be determined from our data if either source is healthier to consume.

## Introduction

By 2050, global food systems will need to meet the dietary demands of almost 10 billion people. To meet these demands in a healthy and sustainable manner, it is put forward that diets would benefit from a shift towards consumption of more plant-based foods and less meat, particularly in Western countries^1^. This has raised questions whether novel plant-based meat alternatives represent healthy and nutritionally adequate alternatives to meat^2–5^.

**Figure 1:**
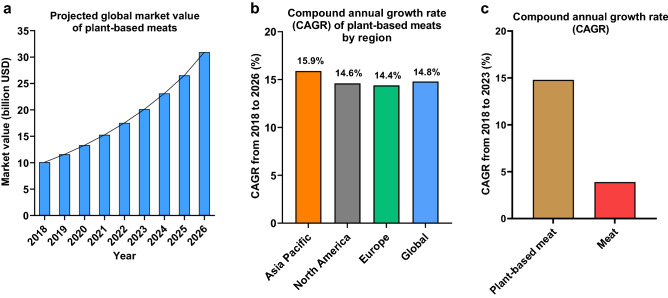
The global market value of plant-based meat alternatives and meat. Market data on plant-based meat alternatives and meat were obtained from STATISTA^7^. (**a**) The projected global market value of plant-based meats from 2018 to 2026 in Billion US Dollars. (**b**) The compound annual growth rate (CAGR) of the plant-based meat sector globally and by region. (**c**) The relative growth of the global plant-based meat sector (+ 14.8%) is expected to exceed the relative growth global animal meat market (+ 3.9%). Despite growth in absolute terms, the value share of the global animal meat sector as a percentage of the overall food industry is expected to remain more or less similar during 2018–2023^7^.

The new generation of plant-based meat alternatives such as the Impossible Burger and Beyond Burger are becoming increasingly popular with consumers. Their success has led other international food companies—including traditional meat companies—to invest in their own product versions^6^. The global plant-based meat alternative sector has experienced substantial growth and is projected to increase from $11.6 billion in 2019 to $30.9 billion by 2026 with a compound annual growth rate (CAGR) of 15% (Fig. 1). In contrast, the meat sector is expecting a CAGR of 3.9% during this time and to reach a market value of $1142.9 billion by 2023^7^.

The production of plant-based meats as a replacement for animal-sourced meat is nothing new. One of the earliest engineered meat alternatives was Protose, a plant-based meat alternative made from wheat gluten, peanuts, and soybean oil, which was designed by John Kellogg in the late nineteenth century. In 1899, Kellogg wrote the following in his patent application for Protose:

“The objective of my invention is to furnish a vegetable substitute for meat which shall possess equal or greater nutritive value in equal or more favorable form for digestion and assimilation and which shall contain the essential nutritive elements in approximately the same proportion as beef and mutton and which substitute has a similar flavor and is as easily digestible as the most tender meat” (U.S. Patent No 670283A).

More than a century later, the objective of plant-based meat alternative production has arguably remained the same; however, contemporary products have arguably accomplished to create a sensory experience that more closely resembles red meat. For example, soy leghemoglobin in a popular plant-based meat alternative imitates the “bloody” appearance and taste of heme proteins in meat, while extracts from red beets, red berries, carrots, and/or other similarly colored vegetables are often embedded in plant-based meat alternatives to give them a reddish “meat-like” appearance^6^. Methyl cellulose is often used to give plant-based meat alternatives a “meat-like” texture, while flavoring agents are added to mimic the taste of cooked meat. Modern meat alternatives also match the protein content of meat by using isolated plant proteins (e.g., soy, pea, potato, mung bean, rice, mycoprotein, and/or wheat), and they are sometimes fortified with vitamins and minerals found in red meat (e.g., vitamins B_12_, zinc, and iron) to provide an even more direct nutritional replacement^4,8^.

**Figure 2:**
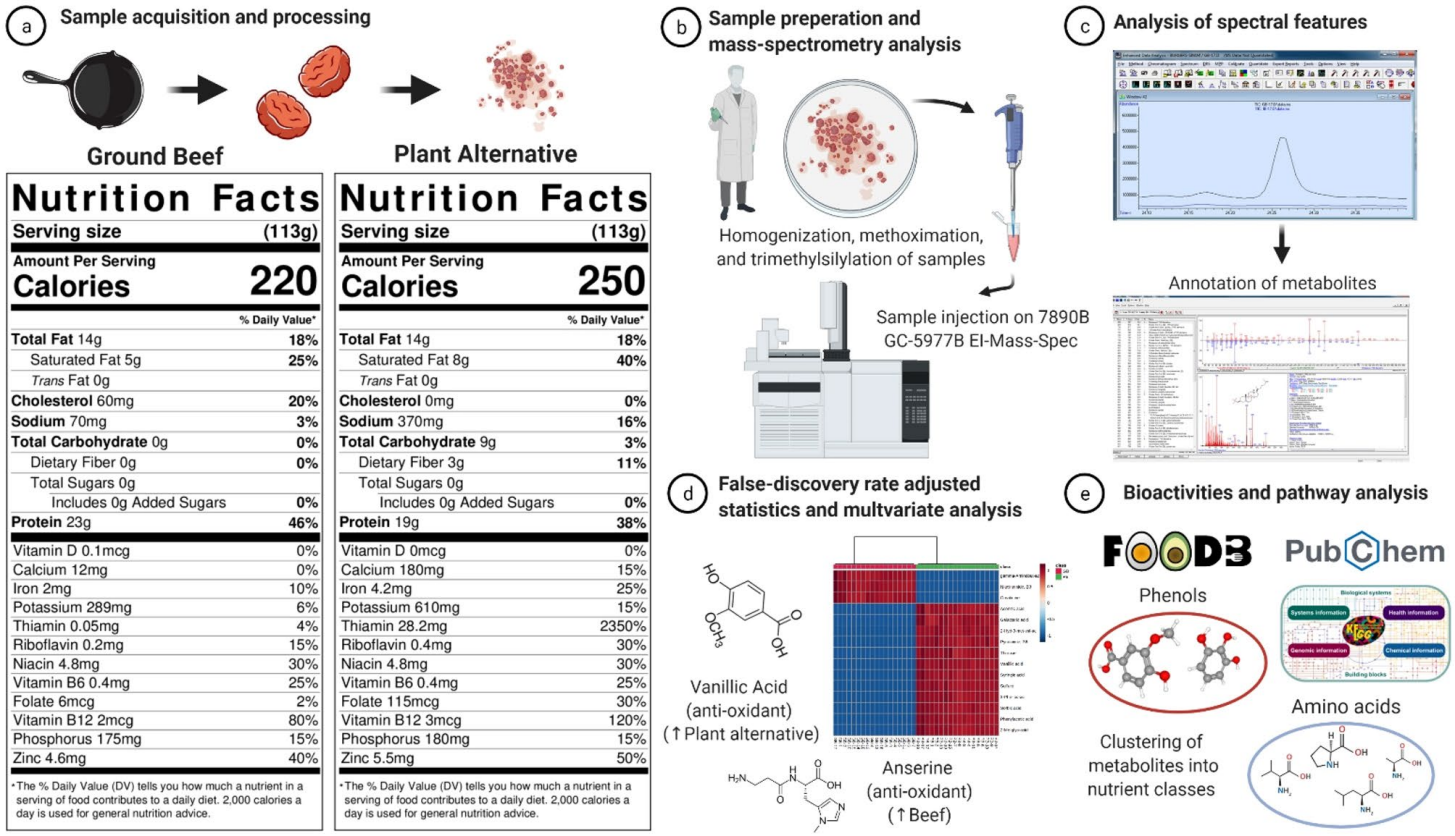
Schematic description of sample preparation and metabolomics analysis. (**a**) Nutrition Facts panels of grass-fed ground beef and the plant-based meat alternative. (**b**) Samples were homogenized, methoximated and trimethylsilylated, and untargeted metabolomic analysis was conducted via gas chromatography/electron-ionization mass spectrometry (GC/EI-MS). (**c**) Raw spectral data were imported into the freeware (Automatic Mass Spectral Deconvolution and Identification Software or AMDIS), and annotated as metabolites using an orthogonal approach that incorporates both retention time (RT) from GC and the fragmentation pattern observed in EI-MS. (**d**) To determine differences in abundance of metabolites between beef and plant-based meat alternative, deconvoluted peak areas from AMDIS analysis were log-base-two transformed prior to analysis and tested using the Wilcoxon rank sum test with Benjamini-Hochberg adjusted p values at 5% (False Discovery Rate adjusted p < 0.05). (**e**) Bioactivities and potential health effects of annotated metabolites were explored using FooDB (https://foodb.ca/) and/or PubChem (https://pubchem.ncbi.nlm.nih.gov/) databases, while metabolic pathway identification of metabolites was performed using the Kyoto Encyclopedia of Genes and Genomes (KEGG) (https://www.genome.jp/)^11^. Figure was created with BioRender.com.

For example, a commercially available plant-based alternative closely matches the Nutrition Facts panel of beef (Fig. 2), and to consumers reading nutritional labels they may appear nutritionally interchangeable^9^. Nonetheless, food sources have considerable complexity and contain a wide variety of nutrients (e.g., phenols, anti-oxidants, peptides, amino acids, fatty acids, biogenic amines etc.), the majority of which do not appear on nutrition labels, but can have potential health implications^10^. Important nutritional differences may exist between beef and novel plant-based alternatives; however, this has not been thoroughly assessed.

Given the scientific and commercial interest in plant-based meat alternatives, the goal of our study was to use untargeted metabolomics to provide an in-depth comparison of the metabolite profiles of grass-fed ground beef and a popular plant-based meat alternative, both of which are sometimes considered as healthier and more environmentally friendly sources of “beef”^4,5,8^. Metabolomics is an analytical profiling technique that allows researchers to measure and compare large numbers of nutrients and metabolites present in biological samples.

## Results

### Product information

A schematic representation of the study flow is provided in Fig. 2. Eighteen different packages (340 g or 12 oz each) of a commercially-available plant-based meat alternative was purchased from a local grocery store in Raleigh, NC, USA. Ground beef from eighteen grass-fed, black angus cattle (454 g or 16 oz each) was purchased from Alderspring Ranch (May, ID) and matched for total fat content to the plant-based alternative, which was confirmed using proximate analysis (method AOAC 960.39; Microbac Laboratories, Warrendale, PA). The macronutrient composition and energy content of 113 g (4oz) grass-fed beef was 24 g of protein, 0 g of carbohydrates, 14 g of fat (5 g saturated fat), and 220 kcal. The macronutrient composition and energy content of 113 g (4oz) the soy-based meat alternative was 19 g of protein, 9 g of carbohydrates, 14 g of fat (8 g saturated fat), and 250 kcal. The Nutrition Facts panels of both food sources, including micronutrients, can be found in Fig. 2. The plant-based alternative is fortified with iron (from soy leghemoglobin), ascorbic acid (vitamin C), thiamin, riboflavin, niacin, vitamin B_6_, vitamin B_12_, and zinc. The micronutrients within grass-fed beef are part of the natural food matrix.

### Metabolomics analysis

We found that a total of 171 out of 190 annotated metabolites (90%) were different (p < 0.05) between beef and the plant-based alternative. Several compounds were found either exclusively (22 metabolites total) or in greater quantities in beef (51 metabolites total) compared with the plant-based meat alternative (all, p < 0.05). Nutrients only found in beef included allantoin (alkaloid; anti-oxidant)^12^, anserine (dipeptide; anti-oxidant)^13^, cysteamine (amine; anti-oxidant)^14^, docosahexaenoic acid (DHA, C22:6, ω-3 essential fatty acid)^15^, glucosamine (hexoamines; collagen biosynthesis)^16^, hydroxyproline (non-protein amino acid; collagen biosynthesis)^17^, gamma-aminobutyric acid (non-protein amino acid; anti-hypertensive)^18^, spermine (biogenic polyamine; anti-oxidant)^19^, niacinamide (vitamin B_3_; neuroprotective)^20^, and squalene (terpenoid phenolic; anti-bacterial)^21^. Several other compounds were found exclusively (31 metabolites total) or in greater quantities (67 metabolites total) in the plant-based meat alternative when compared to beef (Supplemental Table 1) (all, p < 0.05). Nutrients only found in the plant-based meat alternative included 2-hydroxy-3-methylvaleric acid (pentanoic acids; flavor)^22^, 3-hydroxyanthranilic acid (phenolic; anti-oxidant)^23^, aconitic acid (carboxylic acid; flavor)^24^, ascorbate (vitamin C; anti-oxidant)^25^, beta-sitosterol (phytosterol; anti-carcinogenic)^26^, campesterol (phytosterol; anti-carcinogenic)^26^, loganin (terpenoid phenolic; anti-inflammatory)^27^, melezitose or similar disaccharides (flavor)^28^, monolaurin (glyceride; anti-microbial)^29^, phenylacetic acid (phenylacetates; anti-oxidant)^30^, sorbic acid (unsaturated fatty acid; food preservative)^31^, sulfurol (phenolic; flavor)^32^, syringic acid (phenolic; anti-oxidant)^33^, trilaurin (glyceride; anti-microbial)^29^, and tyrosol (phenolic; anti-oxidant)^34^. A full list of annotated metabolites, their respective pathway classification (KEGG-identified)^11^, and their potential bioactivates/health effects (FoodDB and PubChem-identified) can be found in Supplemental Table 1. While several of these nutrients are considered non-essential or conditionally-essential based on life-stages (e.g., infancy, pregnancy, or advanced age) and are often less appreciated in discussions of human nutritional requirements^10^, their importance should not be ignored, as their absence (or presence) can potentially impact human metabolism and health.

**Table 1:** Metabolites clustered into nutrient classes according to structural similarity using ChemRICH software procedures^11^. Arrow (↑) indicates higher abundance for a particular nutrient class or nutrient. Abbreviations used: AA, amino acids; DHA; docosahexaenoic; FA, fatty acid.

Metabolite class	#	↑Plant	↑Beef	p value	Key compound	Metabolic pathway, potential bioactivities
Amino acids	19	13	6	< .001	Glutamine (↑Plant)	Protein metabolism, neurotransmitter
Non-protein AAs	14	5	6	< .001	Creatinine (↑Beef)	Energy metabolism, neuroprotective
Saccharides	13	8	4	< .001	Keto pentose-5-phos (↑Beef)	Energy metabolism, flavor
Saturated FAs	13	5	6	< .001	Pentadecanoic acid (↑Beef)	Odd-chain fatty acid biosynthesis, anti-oxidant
Dicarboxylic acids	11	4	7	< .001	Aminomalonic acid (↑Beef)	Glycine metabolism, unknown
Phenols	8	6	2	< .001	Vanillic acid (↑Plant)	Plant/microbial metabolism, anti-inflammatory
Dipeptides	8	2	4	< .001	Anserine (↑Beef)	Carnosine metabolism, antioxidant
Purines	7	3	4	< .001	Uric acid (↑Beef)	Microbrial/purine metabolism, unknown
Sugar alcohols	7	4	2	< .001	Myoinositol (↑Beef)	Biosynthesis, liver-protective, neuroprotective
Hydroxybuyrates	6	4	2	< .001	4-Hydroxybutyric acid (↑Beef)	Biosynthesis, neuroprotective
Vitamins	5	3	2	< .001	Vitamin C (↑Plant)	Biosynthesis, anti-oxidant, liver-protective
Glycerides	5	4	0	.002	Monolaurin (↑Plant)	Lipid metabolism, anti-inflammatory
Pentoses	4	2	2	< .001	Arabinose/aldopentose (↑Beef)	Energy metabolism, antioxidant, flavor
Sugar acids	4	3	1	< .001	Glyceric acid (↑Beef)	Biosynthesis, cholesterolytic, kidney-protective
Amino alcohols	4	3	1	< .001	Phosphoethanolamine (↑Beef)	Sphingolipid metabolism, neurotransmitter
Pyrimidines	4	1	2	.001	Dihydrouracil (↑Beef)	Pyrimidine metabolism, neuro-protective
Amines	4	0	3	.001	Cysteamine (↑Beef)	Taurine metabolism, antioxidant,
Unsaturated FAs	3	2	2	.003	Sorbic Acid (↑Plant)	Fatty acid biosynthesis, preservative
Phytosterols	3	3	0	.003	Stigmasterol (↑Plant)	Biosynthesis, antioxidant, cancer-protective
Tocopherols	3	3	0	.003	α-Tocopherol (↑Plant)	Biosynthesis, antioxidant, cardio-protective
Biogenic amines	3	2	1	.003	Spermidine (↑Plant)	Glutathione metabolism, antioxidant
Polyunsaturated FAs	3	0	2	.007	DHA, 22-6, ω-3 (↑Beef)	Essential fatty acid, neuroprotective
Pyridines	3	0	2	.014	3-Hydroxypyridine (↑Beef)	Maillard reaction end-product, flavor
Fatty acid esters	3	1	0	1.00	1,2-Dicaprin (↑Plant)	Energy metabolism, biosynthesis

**Figure 3:**
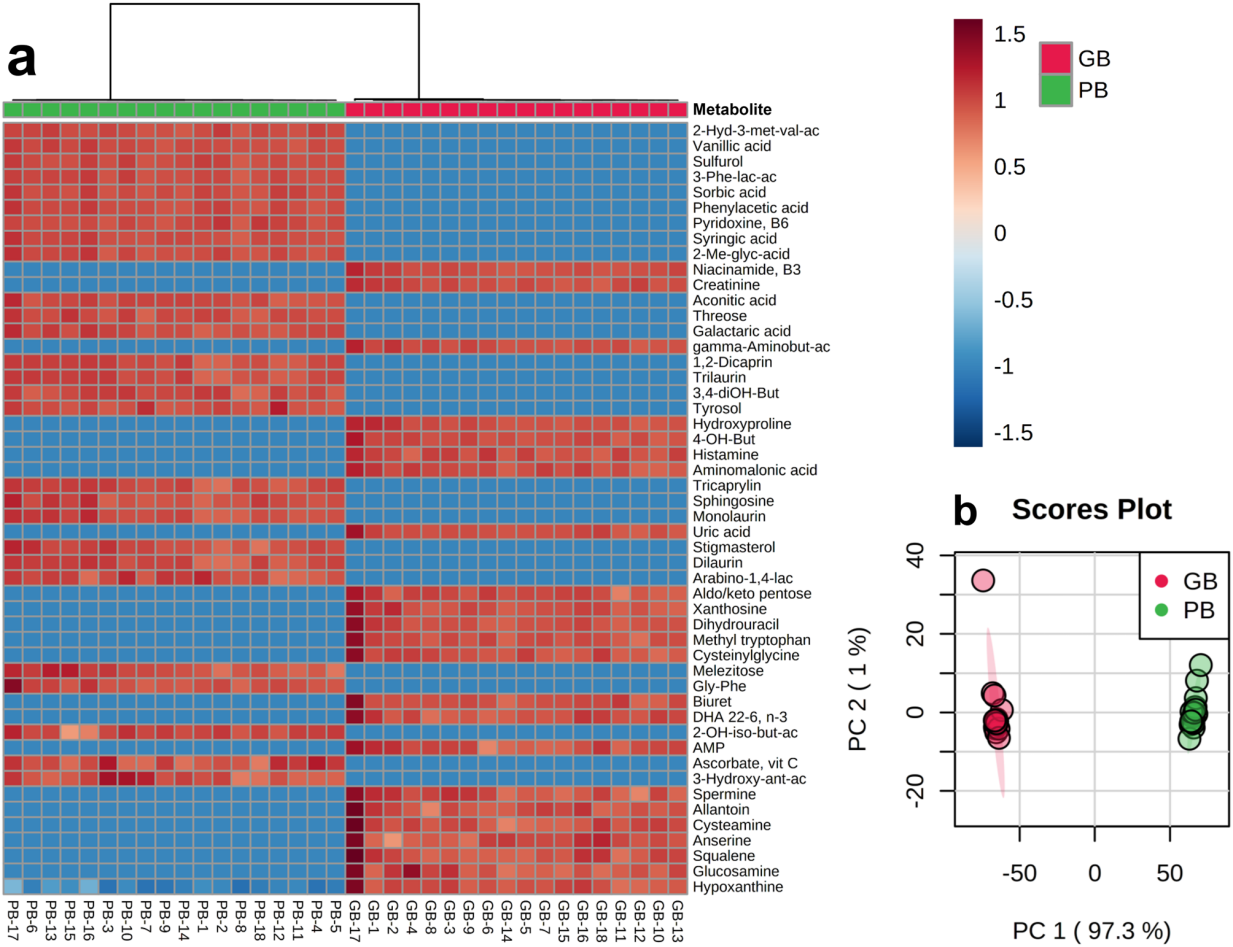
Metabolomics revealed distinct differences in metabolite profiles between grass-fed ground beef (GB) and the plant-based meat alternative (PB). (**a**) Heatmap of the top 50 metabolites, ranked by p values (lowest to highest), that were significantly different (p < 0.05) between beef and the plant-based meat alternative. Red (intensity ranges from 0 to 1.5) means higher abundance of the corresponding metabolite, whereas blue means lower abundance (intensity ranges from − 0 to − 1.5). The numbers below the heatmap represent individual samples (GB-1 to 18 and PB-1 to 18 respectively; *n* = 18 for each group). Metabolites in beef and the plant-based meat alternative were compared by the Wilcoxon rank sum test with Benjamini–Hochberg adjusted p values at 5% (p < 0.05). (**b**) Principal Component Analysis (PCA) analysis of beef and plant-based meat revealed a distinct difference in metabolite composition between the grass-fed ground beef and the plant-based meat, with 97.3% of the variance explained within the first principal component (PC1)—which illustrates that large differences exist between beef and the plant-based meat alternative. Red and green colors above the heatmap (**a**) and the PCA plot (**b**) represent the ground beef and the plant-based meat, respectively. The 95% confidence interval of the groups is depicted in their respective color. A full list of potential bioactivities and health effects of each individual metabolite is reported in Supplemental Table 1. Figure was created with MetaboAnalyst 4.0 (http://www.metaboanalyst.ca)^35^.

To visualize differences and identify the top metabolites contributing to the nutritional disparity between beef and plant-based meat, we created a ranked heatmap of the top fifty metabolites based on the Pearson distance measure and the Ward clustering algorithm, and performed unsupervised principal component analysis using software procedures from MetaboAnalyst 4.0 (http://www.metaboanalyst.ca)^35^. Both the heatmap (Fig. 3A) and unsupervised principal component (PCA) analysis (Fig. 3B) revealed a distinct separation in nutritional components, with 97.3% of the variance explained within the first principal component (PC1)—further illustrating that large metabolite differences exist between beef and the plant-based meat alternative.

### Chemical similarity enrichment analysis

To identify the main metabolite classes that differed between beef and the plant-based alternative, we clustered individual metabolites into metabolite classes according to their structural similarity using Chemical Similarity Enrichment Analysis (ChemRICH) software procedures (http://chemrich.fiehnlab.ucdavis.edu/). We identified 24 classes (enrichment clusters) with ≥ 3 structurally similar metabolites regardless of whether these metabolites were found in beef or the plant-based meat alternative (Table 1). We found that 23 out of the 24 identified metabolite classes differed significantly (false discovery rates adjusted p < 0.05) between beef and the plant-based meat alternative.

**Figure 4:**
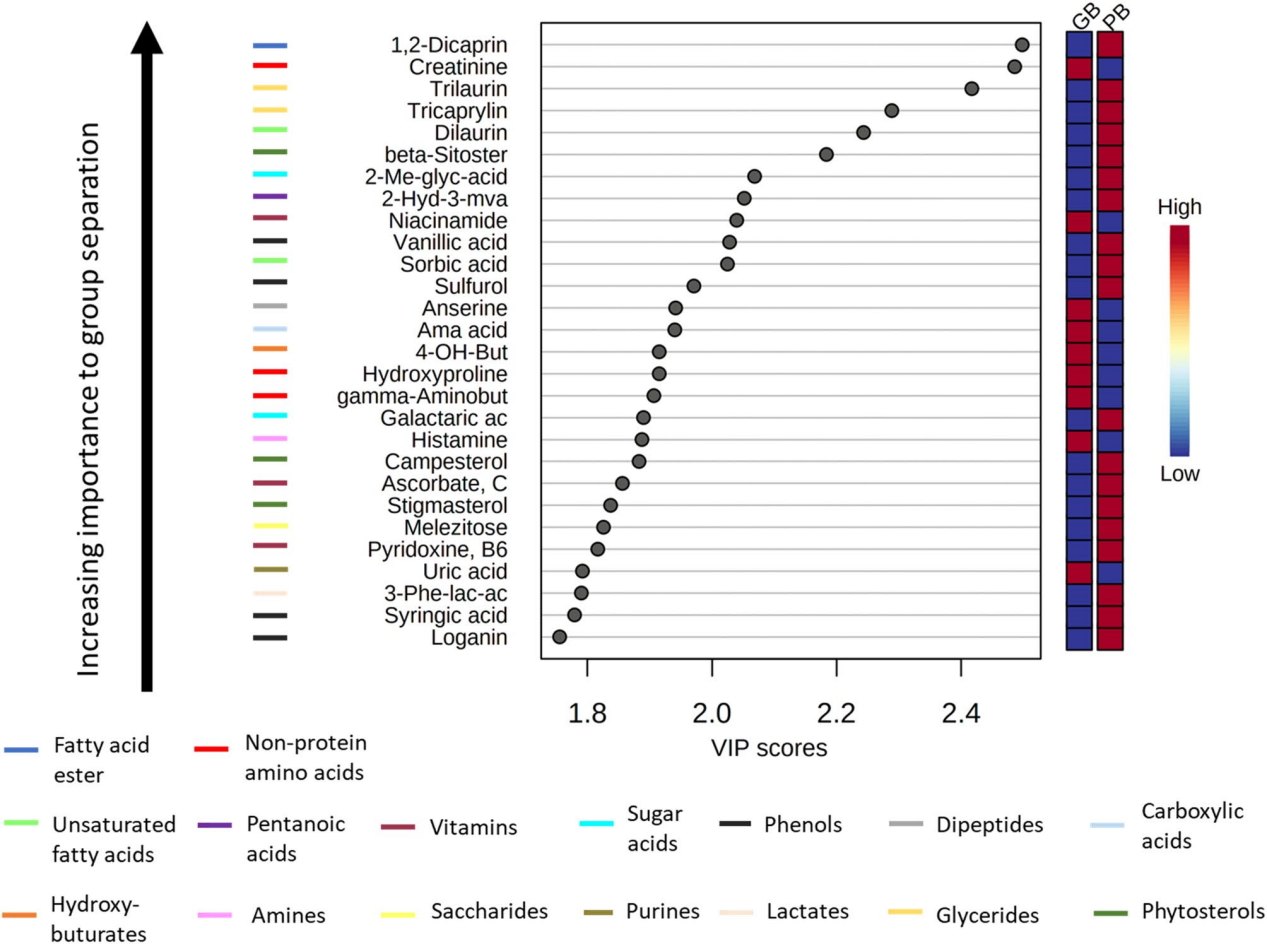
Partial least squares-discriminant analysis (PLS-DA) was used to rank metabolites according to their prognostic importance (VIP scores) in separating the metabolite profiles of beef and the plant-based meat alternative. The boxes on the right of the plot indicates the relative concentrations of the corresponding metabolite in the plant-based meat alternative and beef. Red means higher abundance of the corresponding metabolite, whereas blue means lower abundance. Metabolite classes were identified using Chemical Similarity Enrichment Analysis (ChemRICH) software procedures (http://chemrich.fiehnlab.ucdavis.edu/)^11^ and indicated by the colored bars left of the metabolites.

Amongst the metabolite classes emerging as most discriminating between beef and the plant-based meat alternative were amino acids, non-protein amino acids, saccharides, saturated fatty acids, dicarboxylic acids, phenols, dipeptides, sugar alcohols, vitamins, glycerides, unsaturated fatty acids, and amino alcohols (all, p < 0.05). We then used partial least squares-discriminant analysis (PLS-DA) (Fig. 4) and identified the metabolites responsible for their overall discrimination ability between groups (ranked according to Variable Importance in Projection [VIP] scores).

## Discussion

A new generation of plant-based meat alternatives have recently entered global consumer markets. Novel plant-based meat alternatives are often formulated and marketed to facilitate the replacement of animal-based foods in the diet, both in terms of sensory experience and nutrition. The plant-based meat alternative and grass-fed, beef studied in our work, have largely similar Nutrition Facts panels and may appear nutritionally interchangeable to consumers^9^. Despite these apparent similarities based on Nutrition Facts panels, our metabolomics analysis found that metabolite abundance between the plant-based meat alternative and grass-fed ground beef differed by 90% (171 out of 190 profiled metabolites; p < 0.05). Substantial differences in metabolites within various classes (e.g., amino acids, dipeptides, vitamins, phenols, tocopherols, odd-chain saturated and unsaturated fatty acids, antioxidants) indicate that these products should not be viewed as nutritionally interchangeable. The new information we provide is important for making informed decisions by consumers, and to inform dietary advice by health professionals.

Several metabolites with potentially important regulatory roles in human health were found either exclusively or in greater quantities in beef samples than in the plant-based alternative and vice versa. Creatinine (product of creatine), hydroxyproline (a non-proteinogenic amino acid), anserine (a carnosine metabolite), glucosamine (a saccharide), and cysteamine (an aminothiol) are examples of nutrients only found in beef and appeared as discriminating metabolites within their respective nutrient class (Table 1). These nutrients have potentially important physiological, anti-inflammatory, and/or immunomodulatory roles^17,36^, and low intakes are associated with cardiovascular, neurocognitive, retinal, hepatic, skeletal muscle, and connective tissue dysfunction^17,36^. For example, creatine and anserine were found to provide neurocognitive protection in randomized controlled trials in older adults^37,38^. Cysteamine, a potent antioxidant, also has neuroprotective effects and is a precursor of glutathione—one of the most potent intracellular anti-oxidants^14^. Squalene has potential anti-oxidant, anti-bacterial, and anti-tumor activity^21^, while dietary hydroxyproline and glucosamine can stimulate collagen biosynthesis and are considered important for maintaining the structure and strength of connective tissue and blood vessels^16,17^.

Metabolites in nutrient classes such as phenols, tocopherols, and phytosterols (Table 1) were found exclusively or in greater abundance in the plant-based meat when compared to beef. For instance, the plant-based meat alternative contained more tocopherols (α, γ, and δ)—a class of nutrients with vitamin E activity best known for their anti-oxidant effects^39^. We also found several phytosterols such as *beta*-sitosterol, campesterol, and stigmasterol in the plant-based meat; compounds that collectively may possess anti-oxidant, anti-inflammatory, and/or cancer-protective properties^26^. We also found a wider variety and greater abundance of phenolic compounds in the plant-based alternative when compared to beef (Table 1). Identified compounds include sulfurol, syringic acid, vanillic acid, and methylated/hydroxylated forms of valeric acid, which may benefit human health by dampening oxidative stress and inflammation^40^. Lastly, we found higher amounts of spermidine in the plant-based meat alternative, which is a biogenic polyamine that has been studied for its potential neurocognitive and cardiovascular benefits^41^.

Within the nutrient class of polyunsaturated fatty acids (PUFAs), the fatty acids arachidonic acid (ARA, C20:4, ω-6) and docosahexaenoic acid (DHA, C22:6, ω-3) were found exclusively (DHA) or in greater quantities (ARA) in grass-fed beef samples (Table 1). These essential fatty acids are major constituents of the brain phospholipid membrane and have important roles in cognition, immunomodulation, platelet function, and cell signaling^15,36^. Their deficiencies are associated with cognitive decline and increased risk of cardiovascular disease^15,36^.

Important differences were also observed in saturated fatty acid and glyceride classes (Table 1). The main saturated fatty acids and glycerides (Table 1) in the plant-based meat were coconut oil-derived lauric acid and monolaurin, which may possess anti-microbial and/or anti-inflammatory properties^29,42^. On the other hand, we found higher levels of the dietary odd-chain saturated fatty acids (OCFAs) pentadecanoic acid (C15:0) and heptadecanoic acid (C17:0) in beef than in the plant-based alternative. These compounds are believed to exert their beneficial effects by attenuating inflammation, dyslipidemia, and cell fibrosis^43^, and increased dietary intake is associated with a lower risk of metabolic disease^44,45^. Other notable metabolites in the plant-based meat alternative were the vitamins niacin (B_3_), pyridoxine (B_6_), and ascorbic acid (C), which are added as fortificants to the product.

When considering the health effects of foods there is a need to appreciate the complexity of whole food sources beyond simply their protein, fat, vitamin, and mineral content^46^. Considerations of how diet affects health is generally limited to 150 nutritional components routinely tracked in nutritional databases of which only 13 routinely appear on nutritional labels (e.g., fat, saturated fat, trans fat, cholesterol, sodium, carbohydrate, fiber, sugar, protein, vitamin A, vitamin C, calcium, and iron)^10^. These nutrients represent only a small fraction of the more than 26,000 metabolites in the ‘human foodome’—many of which have potential health effects but often remain underappreciated in discussions of diet and human health^10,46^. The complexity of the whole food matrix—as indicated here by our metabolomics findings—highlights that attempting to mimic food sources using single constituents such as isolated proteins, vitamins, and minerals is challenging and arguably underestimates the complexity of the food source it is meant to mimic.

While additional fortification and technological advancement could potentially somewhat increase the nutritional similarity of plant-based meat alternatives and meat, since foods contain thousands of constituents that can synergistically impact human metabolism, consuming isolated nutrients or fortified foods often do not confer similar benefits when compared to ingesting nutrients as part of their whole-food matrix^47^. For example, supplementation of a low-meat diet with zinc and other minerals found in meat did not result in a similar in vivo zinc status when equivalent amounts of these minerals were provided as part of the natural meat matrix^48^. Similar findings have been made for other nutrients, such as copper, calcium and vitamins A, C, and D, which are associated with disease protection when obtained from food, but often not when obtained from fortified or supplemental sources^49,50^.

The uptake of minerals such as iron^51^ and zinc^52^ is also reduced when obtained from legumes (the common protein source in plant-based meat alternatives) when compared to animal foods. That is because iron in animal foods is found in the heme form, which is more bioavailable than the non-heme form found in plant foods^53^. Of note is that the iron in a novel plant-based meat alternative is also present in the heme form, and is produced from *Pichia pastori* yeast encoded with the leghemoglobin protein gene that is normally expressed in root nodules of soy plants (i.e., soy leghemoglobin)^54^. Work performed in an *in vitro* human epithelial model found that iron uptake from leghemoglobin is comparable to bovine hemoglobin^55^, and future work is needed to confirm the potential ability of soy leghemoglobin in plant-based meat alternatives to contribute to iron status *in vivo* in humans. Uptake of zinc from plant foods is also reduced due to the presence of phytates, lectins, and other anti-nutrients^52^, but can be improved when consumed at higher meal-like protein levels^56^, which is the case for novel plant-based meat alternatives given their use of isolated plant proteins. For an in-depth discussion on potential nutrient uptake from meat and plant-based meat alternatives we refer to a recent publication^5^.

Similarities in terms of total protein content (~20 g protein) and several vitamins/minerals (e.g., zinc, iron, vitamin B_12_, and niacin) between beef and the plant-based alternative, studied in this work (Fig. 2), does indicate that a “flexitarian approach” (replacing limited amounts of animal foods with plant-based alternatives) is unlikely to negatively impact essential nutritional status of consumers, but this also depends on what other foods are part of the diet and the degree to which plant-based substitutes replace animal foods (e.g., the occasional replacement or full replacement). Caution is warranted for vulnerable populations such as children, women of childbearing age, and older individuals who may be at increased risk for suboptimal nutritional status with low intakes of animal foods^57,58^.

Plant-based diets are commonly associated with higher dietary quality and lower chronic metabolic disease risk when compared to typical omnivorous diets^59–61^. Several prospective studies have found that consumption of plant foods such as legumes, whole grains, and nuts is associated with lower cancer rates, cardiovascular disease risk, and all-cause mortality, whereas red and processed meat is generally associated with an elevated risk^62,63^, particularly when consumed as part of Standard American/Western diets^64–66^. It must be noted that in the context of healthful dietary patterns, plant-based (vegan/vegetarian) and omnivorous diets may be equally associated with lower chronic metabolic disease risk and/or mortality^67–70^.

While plant foods are generally considered healthy to consume, Hu and colleagues^2^ have expressed reservation in extending these notions to several novel plant-based meat alternatives given their ultra-processed nature. Not all plant-based diets are necessarily healthy either, and diets rich in ultra-processed foods are associated with increased chronic metabolic disease risk, irrespective of whether they are plant-based or mixed (omnivorous)^71,72^. Whether several novel plant-based meat alternatives fit the description of a desirable plant food in healthy dietary patterns is currently debated^2,4,5,73^, and likely depends on product formulations, frequency of consumption, and the background diet of the individual. Further work is needed to inform these discussions; however, we consider it important to not lose sight of the “bigger picture” in these discussions, which is the overall dietary pattern in which individual foods are consumed. That is arguably the predominant factor dictating health outcomes to individual foods^74^. Of note is a recent 8-week randomized controlled trial that found that a “flexitarian approach” (swapping moderate amounts of meat with novel plant-based alternatives as part of an omnivorous diet) may have positive benefits in terms of weight control and lipoprotein profiles (e.g., LDL-cholesterol)^73^.

Our work has limitations. While the plant-based meat alternative studied here is one of the most popular products currently on the market, product formulations of novel plant-based meat alternatives differ in terms of the type of isolated plant proteins (e.g., soy, pea, potato, mung bean, rice, mycoprotein and/or wheat), fats (e.g., canola, soy, coconut, and/or sunflower oil), and/or other novel ingredients (e.g., soy leghemoglobin, different vegetable extracts, and/or different flavoring agents)^8^. Furthermore, we used GC-MS for metabolic profiling and it is possible that other platforms (e.g., LC-MS) may reveal additional differences or similarities in metabolite profiles. Nonetheless, our metabolomics analysis and a recent comparison of fatty acids and volatile (flavor) compounds in other plant-based meat alternatives with a beef burger suggests that, in general, plant-based meat alternatives are substantially different from animal meat when considering expanded nutritional profiles^75^. As the field of food-omics (the application of metabolomics in food and nutrition sciences) progresses, we will undoubtedly gain further appreciation of the complexity of natural food matrices and the ability of manifold nutritional constituents to synergistically modulate human health^10^.

In conclusion, metabolomics revealed that abundance of 171 out of 190 profiled metabolites differed between beef and a commercially-available plant-based meat alternative, despite comparable Nutrition Facts panels. Amongst identified metabolites were various nutrients (amino acids, phenols, vitamins, unsaturated fatty acids, and dipeptides) with potentially important physiological, anti-inflammatory, and/or immunomodulatory roles—many of which remained absent in the plant-based meat alternative when compared to beef and vice versa. Our data indicates that these products should not be viewed as nutritionally interchangeable, but could be viewed as complementary in terms of provided nutrients. It cannot be determined from our data if either source is healthier to consume. Just a peanut is not really an egg^76^, we conclude that a plant burger is not really a beef burger. Thus, our work adds to the notion that caution is warranted when categorizing foods as equivalent for consumers simply based on their protein content (“protein foods”), which is typical in dietary recommendations^76,77^.

Future studies are needed to better understand how the presence and absence of metabolites and nutrients in plant-based meat alternatives and meat impacts short- and long-term consumer health. Studies performed in various populations (children, elderly, those with metabolic disease etc.), and in response to various types and amounts of plant-based meat alternatives are required to evaluate their healthfulness and appropriateness within the human diet.

## Materials and methods

### Sample preparation

Individual patties (113 g or 4 oz each) were formed from each package of plant-based meat (*n* = 18) and beef (*n* = 18), respectively. Individual patties were cooked in a non-stick skillet until the internal temperature of each patty read 71 °C as determined by a meat thermometer. One-gram microcore samples were obtained from the middle of each patty using a bioptome device, immediately frozen in liquid nitrogen, and stored at − 80 °C until metabolomics analysis. Microcore samples the plant-based meat replacement and bovine skeletal muscle (i.e., beef) were powdered under liquid N2 and homogenized in 50% aqueous acetonitrile containing 0.3% formic acid (50 mg wet weight sample per ml homogenate) using a Qiagen Retsch Tissue Lyser II set to a frequency of 30 oscillations/s for a total of 2 min with one 5 mm glass ball (GlenMills, Inc, #7200-005000TM) per tube. 100 µl of each sample homogenate was then transferred into a fresh, 1.5-ml, Reduced Surface Activity (RSATM) glass autosampler vial (catalog number 9512C-1MP-RS, MicroSolv Technology Corporation, Leland, NC). Proteins in sample homogenates were subsequently “crash" precipitated with 750 µl dry methanol and centrifuged at 13.500×*g* rcf for 5 min (Vial Centrifuge^TM^, MicroSolv, catalog C2417). The crash solvent was spiked with D_27_-deuterated myristic acid (D27-C14:0) (Sigma 366889, 6.25 mg/l) an internal standard for retention-time locking (described below). 700 µl of the supernatant of each sample homogenate was subsequently transferred to fresh RSATM glass vials (catalog number 9512C-1MP-RS, MicroSolv Technology Corporation, Leland, NC). Methanolic extracts were then dried in a Savant SPD111V SpeedVac Concentrator (Thermo Scientific, Asheville, NC), with the help of a final pulse of toluene (Fisher Scientific, catalog number T324-50) as an azeotropic drying agent. 25 µl methoxyamine hydrochloride (18 mg/ml in dry pyridine: Fisher Scientific, catalog number T324-50) was then added to each sample and incubated at 50 °C for 30 min for methoximation of certain reactive carbonyl groups. Finally, metabolites were rendered volatile by replacement of easily exchangeable protons with trimethylsilyl (TMS) groups using *N*-methyl-*N*-(trimethylsilyl) trifluoroacetamide (MSTFA; 75 µl per sample Cerilliant M-132, Sigma, St. Louis, MO) at 50 °C for 30 min.

### GC/EI-MS analysis

Biological comparators (beef vs. plant-meat based alternative) were run in direct succession (e.g., the order A-B-A-B) on a 7890B GC/5977B single-quadrupole, Inert MS (Agilent Technologies, Santa Clara, CA). This system was equipped with a MultiMode Inlet (MMI), which, in combination with a mid-column, purged ultimate union (PUU), enabled hot back-flushing of the upstream half of the column at the end of each run to reduce fouling of both GC and MS with heavy contaminants (“high boilers”) and carryover between injections. Briefly, the two wall-coated, open-tubular (WCOT) GC columns connected in series were both from J&W/Agilent (part 122-5512 UI), DB5-MS UI, 15 m in length, 0.25 mm in diameter, with a 0.25-µm luminal film. This film is a nonpolar, thermally stable, phenyl-arylene polymer, similar in performance to traditional 5%-phenyl-methylpolysiloxane films. Prior to each daily run (2 total), the starting inlet pressure was empirically adjusted such that the retention time of the TMS-D27-C14:0 standard is set at ~ 16.727 min. Following an initial distillation within the MMI, the GC oven ramps from 60 to 325 °C at a speed of 10 °C/min. Under these conditions, derivatized metabolites elute from the column and reach the MS detector at known times (e.g., bis-TMS-lactic acid at ~ 6.85 min, and TMS-cholesterol at ~ 27.38 min) within specific tolerance of < 0.1 min. Radical cations generated with conventional electron ionization via a tungsten-rhenium filament set to an energy of 70 eV were scanned broadly from 600 to 50 m/z in the detector throughout the run. Cycle time was approximately 38 min. Our GC/MS methods are based on validated methods and follow those reported by Roessner et al.^78^, Fiehn et al.^79^, Kind et al.^80^, McNulty et al.^81^, Banerjee et al.^82^, and Clinton et al.^83^.

### Data reduction

Raw data from Agilent's MassHunter software environment were imported into the freeware, Automatic Mass Spectral Deconvolution and Identification Software or AMDIS (version 2.73)—^84–86^; courtesy of NIST (http://chemdata.nist.gov/mass-spc/amdis/). Peaks were not normalized, with all samples run in a single batch sequence, for which we have found normalization of peak intensities to not be necessary. Deconvoluted spectra were annotated as metabolites using an orthogonal approach that incorporates both retention time (RT) from GC and the fragmentation pattern observed in EI-MS. Peak annotation was based primarily on our own RT-locked spectral library of metabolites (2059 spectra from 1174 unique compounds at the time of analysis; January 2020). Our library is built upon the Fiehn GC/MS Metabolomics RTL Library (a gift from Agilent, their part number G1676-90000; Kind et al. 2009^80^. Additional spectra have been gleaned from running pure reagent standards in our lab, from the Golm Metabolome Library^87^ (http://csbdb.mpimp-golm.mpg.de/csbdb/gmd/gmd.html), and from the Wiley 10th-NIST 2014 commercial library (Agilent G1730-64000). Peak alignment and chemometrics of log-base-two-transformed areas of deconvoluted peaks were performed with our own custom macros, written in our lab in Visual Basic (version 6.0) for use in the Excel (Microsoft Office Professional Plus 2019) software environment (both from Microsoft, Redmond, WA).

### Data processing

Three investigators (S.V.V., J.R.B., and M.J.M.) subsequently performed line-by-line manual curation to fix miscalls and highlighted ambiguities inherent in certain isomeric or otherwise similar metabolites. Metabolites were retained for further analysis if detected in ≥ 80% of samples of either the plant-based meat replacement or ground beef (i.e., 14 out of 18 samples per group). If a discernable signal for a biochemical was observed in ≥ 80% of samples of one group (beef or plant), but remained absent in all samples of the other group, we assumed absence of meaningful amounts and imputed a small value close to 1 prior to log-base-two-transformation. As can be observed from Supplemental Table 1, this was the case for 53 metabolites, which were readily detected in one source (e.g., beef or the plant-based alternative) but not the other.

This decision was made after careful deliberation with a biostatistician (C.F.P.) and the Metabolomics Core at the Duke Molecular Physiology Institute, and was based on the expectation that in such cases the metabolite feature was truly nonexistent (or at least below the Level of Detection) for a given group (beef or plant-based meat alternative) and not due to chromatographic non-detection. In other words, had the metabolite been present in the food source at meaningful levels, it would have registered as we detected this metabolite in ≥ 80% of samples in the other group (i.e., 14 out of 18 samples).

To illustrate this with an example; anserine (β-alanyl-1-methyl-l-histidine; a methylated product of carnosine) is metabolite that is well-known to occur in beef and other animal meats, but known to be absent in plant samples^17^. Similarly, isoflavones such as β-sitosterol and campesterol would normally not be found in grass-fed beef, but were readily detected in all plant-based meat samples. If we used-nearest neighbor (KNN) imputation (or other commonly used imputation methods such as Partial Least Squares, Singular Value Decomposition, Bayesian Principal Component Analysis etc.) without accounting for true absence of metabolites in a given group, our data set would falsely imply that some metabolites are in the plant or beef source of which their presence is implausible. In the case of remaining missing values for other metabolites, for which a signal was detected in ≥ 14 of samples of one group (beef or plant) and ≥ 1 sample of the other group, k-nearest neighbor imputation was performed^88,89^. The full list of annotated metabolites and their retention times are presented in Supplemental Table 2.

### Data analysis

After data processing, individual metabolites were tested for normality using Kolmogorov-Smirnov tests (p < 0.05) using SAS 9.4 (Cary, North Carolina, USA). Several metabolites did not show a normal distribution after log transformation, which may be expected based on the observed large differences in metabolites abudance between beef and the plant-based meat alternative—53 metabolites were detected exclusively in either the plant-based meat alternative or beef. To test differences in individual metabolites between groups, we subsequently used the non-parametric Wilcoxon with Benjamini–Hochberg adjusted p values at 5% to account for false discovery in our statistical analysis (FDR adjusted p < 0.05).

To visualize differences in individual metabolites between groups and identify the top metabolites that contributed to the nutritional differences between beef and the plant-based meat alternative, we created a ranked heatmap of the top fifty metabolites based on the Pearson distance measure and the Ward clustering algorithm and performed unsupervised principal component analysis using software procedures from MetaboAnalyst 4.0 (https://www.metaboanalyst.ca) (Fig. 3)^35^. Partial Least Square-Discriminant Analysis (PLS-DA) was used to determine the variable importance in projection (VIP) of each compound. The VIP plot generated from the PLS-DA models ranks individual metabolites for their ability to discriminate the grass-fed beef from the plant-based meat alternative and those with score ≥ 1.75 are reported in Fig. 4.

Potential bioactivities and health effects of annotated metabolites were explored by entering Chemical Abstracts Service (CAS) # of individual metabolites in FooDB (https://foodb.ca/) and/or PubChem (https://pubchem.ncbi.nlm.nih.gov/) databases, while metabolic pathway identification of individual metabolites was performed using the Kyoto Encyclopedia of Genes and Genomes (KEGG) (https://www.genome.jp/)^11^. To inform discussions, we clustered metabolites by chemical class using freely-available ChemRICH software procedures (http://chemrich.fiehnlab.ucdavis.edu/)^90^. To enable cluster analysis via structural similarity and ontology mapping, InChiKeys, PubChemID and SMILES canonicals for each metabolite was retrieved by entering its respective Chemical Abstracts Service (CAS) # in the PubChem database (https://pubchem.ncbi.nlm.nih.gov/).

After ChemRICH analysis, the lead investigator (S.V.V.) performed line-by-line manual curation to fix any apparent miscalls or apparent misclassification of individual metabolites, and to perform manual adjustment of metabolite classification when appropriate, after which analysis was re-ran (e.g., ChemRICH classified pyrodixine as a separate “Vitamin B_6_” category in which case the metabolite was subsequently lumped into a larger class labeled “Vitamins”).

## Supplementary information

Supplementary Table 1.

Supplementary Table 2.

## References

[1] Godfray HCJ (2018). Meat consumption, health, and the environment. Science.

[2] Hu FB, Otis BO, McCarthy G (2019). Can plant-based meat alternatives be part of a healthy and sustainable diet?. JAMA.

[3] Godfray, H. C. J. Meat: The future series—Alternative proteins. *World Economic Forum, Geneva, Switzerland*, http://www3.weforum.org/docs/WEF_White_Paper_Alternative_Proteins.pdf. Accessed 24 July 2020 (2019).

[4] Curtain F, Grafenauer S (2019). Plant-based meat substitutes in the flexitarian age: An audit of products on supermarket shelves. Nutrients.

[5] Van Vliet S, Kronberg SL, Provenza FD (2020). Plant-based meats, human health, and climate change. Front. Sust. Food. Syst..

[6] Sha L, Xiong YL (2020). Plant protein-based alternatives of reconstructed meat: Science, technology, and challenges. Trends. Food. Sci. Technol..

[7] STATISTA. Meat substitutes market in the U.S. https://www.statista.com/ (2020).

[8] Bohrer BM (2019). An investigation of the formulation and nutritional composition of modern meat analogue products. Food Sci. Hum. Well..

[9] International Food Council. A consumer survey on plant alternatives to animal meat. https://foodinsight.org/wp-content/uploads/2020/01/IFIC-Plant-Alternative-to-Animal-Meat-Survey.pdf (2020).

[10] Barabási A-L, Menichetti G, Loscalzo J (2020). The unmapped chemical complexity of our diet. Nat. Food.

[11] Kanehisa M (2019). Toward understanding the origin and evolution of cellular organisms. Protein Sci..

[12] Shestopalov AV (2006). Biological functions of allantoin. Biol. Bull..

[13] Kohen R, Yamamoto Y, Cundy KC, Ames BN (1988). Antioxidant activity of carnosine, homocarnosine, and anserine present in muscle and brain. Proc. Natl. Acad. Sci. U.S.A..

[14] Paul BD, Snyder SH (2019). Therapeutic applications of cysteamine and cystamine in neurodegenerative and neuropsychiatric diseases. Front. Neurol..

[15] Ruxton CHS, Reed SC, Simpson MJA, Millington KJ (2004). The health benefits of omega-3 polyunsaturated fatty acids: A review of the evidence. J. Hum. Nutr. Diet..

[16] Kumar MNVR, Muzzarelli RAA, Muzzarelli C, Sashiwa H, Domb AJ (2004). Chitosan chemistry and pharmaceutical perspectives. Chem. Rev..

[17] Wu G (2020). Important roles of dietary taurine, creatine, carnosine, anserine and 4-hydroxyproline in human nutrition and health. Amino Acids.

[18] Briguglio M (2018). Dietary neurotransmitters: A narrative review on current knowledge. Nutrients.

[19] Løvaas E, Carlin G (1991). Spermine: An anti-oxidant and anti-inflammatory agent. Free Radic. Biol. Med..

[20] Fricker RA, Green EL, Jenkins SI, Griffin SM (2018). The influence of nicotinamide on health and disease in the central nervous system. Int. J. Tryptophan Res..

[21] Reddy LH, Couvreur P (2009). Squalene: A natural triterpene for use in disease management and therapy. Adv. Drug Deliv. Rev..

[22] Wang X, Guo M, Song H, Meng Q, Guan X (2021). Characterization of key odor-active compounds in commercial high-salt liquid-state soy sauce by switchable GC/GC × GC–olfactometry–MS and sensory evaluation. Food Chem..

[23] Leipnitz G (2007). In vitro evidence for an antioxidant role of 3-hydroxykynurenine and 3-hydroxyanthranilic acid in the brain. Neurochem. Int..

[24] Tawaraya K (2014). Metabolite profiling of soybean root exudates under phosphorus deficiency. Soil Sci. Plant. Nutr..

[25] Frei B, England L, Ames BN (1989). Ascorbate is an outstanding antioxidant in human blood plasma. Proc. Natl. Acad. Sci. U.S.A..

[26] Othman RA, Moghadasian MH (2011). Beyond cholesterol-lowering effects of plant sterols: clinical and experimental evidence of anti-inflammatory properties. Nutr. Rev..

[27] Yamabe N (2010). Evaluation of loganin, iridoid glycoside from Corni Fructus, on hepatic and renal glucolipotoxicity and inflammation in type 2 diabetic db/db mice. Eur. J. Pharmacol..

[28] Côté GL (2007). Novel Enzyme Technology for Food Applications.

[29] Kabara JJ (1984). Antimicrobial agents derived from fatty acids. J. Am. Oil Chem. Soc..

[30] Chen J (2020). Structure-antioxidant activity relationship of methoxy, phenolic hydroxyl, and carboxylic acid groups of phenolic acids. Sci. Rep..

[31] Piper JD, Piper PW (2017). Benzoate and sorbate salts: A systematic review of the potential hazards of these invaluable preservatives and the expanding spectrum of clinical uses for sodium benzoate. Compr. Rev. Food Sci. Food Saf..

[32] Goodner K, Rouseff R (2011). Practical Analysis of Flavor and Fragrance Materials.

[33] Vo QV (2020). Theoretical and experimental studies of the antioxidant and antinitrosant activity of syringic acid. J. Organ. Chem..

[34] Karković Marković A, Torić J, Barbarić M, Jakobušić Brala C (2019). Hydroxytyrosol, tyrosol and derivatives and their potential effects on human health. Molecules.

[35] Chong J, Wishart DS, Xia J (2019). Using MetaboAnalyst 4.0 for comprehensive and integrative metabolomics data analysis. Curr. Protoc. Bioinformatics.

[36] Tallima H, El Ridi R (2018). Arachidonic acid: Physiological roles and potential health benefits—A review. J. Adv. Res..

[37] Rokicki J (2015). Daily carnosine and anserine supplementation alters verbal episodic memory and resting state network connectivity in healthy elderly adults. Front. Aging Neurosci..

[38] Avgerinos KI, Spyrou N, Bougioukas KI, Kapogiannis D (2018). Effects of creatine supplementation on cognitive function of healthy individuals: A systematic review of randomized controlled trials. Exp. Gerontol..

[39] Fang YZ, Yang S, Wu GY (2002). Free radicals, antioxidants, and nutrition. Nutrition.

[40] Márquez Campos E, Stehle P, Simon M-C (2019). Microbial metabolites of flavan-3-ols and their biological activity. Nutrients.

[41] Madeo F, Eisenberg T, Pietrocola F, Kroemer G (2018). Spermidine in health and disease. Science.

[42] Dayrit FM (2015). The properties of lauric acid and their significance in coconut oil. J. Am. Oil Chem. Soc..

[43] Venn-Watson S, Lumpkin R, Dennis EA (2020). Efficacy of dietary odd-chain saturated fatty acid pentadecanoic acid parallels broad associated health benefits in humans: could it be essential?. Sci. Rep..

[44] Forouhi NG (2014). Differences in the prospective association between individual plasma phospholipid saturated fatty acids and incident type 2 diabetes: the EPIC-InterAct case-cohort study. Lancet Diabetes Endocrinol..

[45] Liu S, van der Schouw YT, Soedamah-Muthu SS, Spijkerman AM, Sluijs I (2019). Intake of dietary saturated fatty acids and risk of type 2 diabetes in the European Prospective Investigation into Cancer and Nutrition-Netherlands cohort: associations by types, sources of fatty acids and substitution by macronutrients. Eur. J. Nutr..

[46] Fardet A, Rock E (2018). Perspective: Reductionist nutrition research has meaning only within the framework of holistic and ethical thinking. Adv. Nutr..

[47] Jacobs DR, Tapsell LC (2007). Food, not nutrients, is the fundamental unit in nutrition. Nutr. Rev..

[48] Hunt JR, Gallagher SK, Johnson LK, Lykken GI (1995). High- versus low-meat diets: Effects on zinc absorption, iron status, and calcium, copper, iron, magnesium, manganese, nitrogen, phosphorus, and zinc balance in postmenopausal women. Am. J. Clin. Nutr..

[49] Xiao Q (2013). Dietary and supplemental calcium intake and cardiovascular disease mortality: The National Institutes of Health–AARP Diet and Health Study. JAMA Intern. Med..

[50] Chen F (2019). Association among dietary supplement use, nutrient intake, and mortality among U.S. adults: A cohort study. Ann. Intern. Med..

[51] Hurrell RF (1992). Soy protein, phytate, and iron absorption in humans. Am. J. Clin. Nutr..

[52] Welch, R. M. in *Zinc in Soils and Plants: Proceedings of the International Symposium on ‘Zinc in Soils and Plants’ held at The University of Western Australia, 27–28 September, 1993* (ed A. D. Robson) 183–195 (Springer, 1993).

[53] Hurrell R, Egli I (2010). Iron bioavailability and dietary reference values. Am. J. Clin. Nutr..

[54] Fraser RZ, Shitut M, Agrawal P, Mendes O, Klapholz S (2018). Safety evaluation of soy leghemoglobin protein preparation derived from *Pichia pastoris*, intended for use as a flavor catalyst in plant-based meat. Int. J. Toxicol..

[55] Proulx AK, Reddy MB (2006). Iron bioavailability of hemoglobin from soy root nodules using a Caco-2 cell culture model. J. Agric. Food. Chem..

[56] Sandström B, Almgren A, Kivistö B, Cederblad Å (1989). Effect of protein level and protein source on zinc absorption in humans. J. Nutr..

[57] Adesogan AT, Havelaar AH, McKune SL, Eilittä M, Dahl GE (2020). Animal source foods: Sustainability problem or malnutrition and sustainability solution? Perspective matters. Glob. Food. Secur..

[58] Phillips SM (2015). Commonly consumed protein foods contribute to nutrient intake, diet quality, and nutrient adequacy. Am. J. Clin. Nutr..

[59] Satija A (2016). Plant-based dietary patterns and incidence of type 2 diabetes in US men and women: Results from three prospective cohort studies. PLoS Med.

[60] Key TJ (2014). Cancer in British vegetarians: updated analyses of 4998 incident cancers in a cohort of 32,491 meat eaters, 8612 fish eaters, 18,298 vegetarians, and 2246 vegans. Am. J. Clin. Nutr..

[61] Kim H (2019). Plant-based diets are associated with a lower risk of incident cardiovascular disease, cardiovascular disease mortality, and all-cause mortality in a general population of middle-aged adults. JAMA.

[62] Zhong VW (2021). Protein foods from animal sources, incident cardiovascular disease and all-cause mortality: A substitution analysis. Int. J. Epidemiol..

[63] Schwingshackl L (2017). Food groups and risk of all-cause mortality: A systematic review and meta-analysis of prospective studies. Am. J. Clin. Nutr..

[64] Kappeler R, Eichholzer M, Rohrmann S (2013). Meat consumption and diet quality and mortality in NHANES III. Eur. J. Clin. Nutr..

[65] Schulze MB, Manson JE, Willett WC, Hu FB (2003). Processed meat intake and incidence of Type 2 diabetes in younger and middle-aged women. Diabetologia.

[66] Grosso G (2017). Health risk factors associated with meat, fruit and vegetable consumption in cohort studies: A comprehensive meta-analysis. PLOS ONE.

[67] Key TJ (2003). Mortality in British vegetarians: Review and preliminary results from EPIC-Oxford. Am. J. Clin. Nutr..

[68] Mihrshahi S (2017). Vegetarian diet and all-cause mortality: Evidence from a large population-based Australian cohort—The 45 and up study. Prev. Med..

[69] Shan Z (2020). Association between healthy eating patterns and risk of cardiovascular disease. JAMA Intern. Med..

[70] Salomé M (2021). Contrary to ultra-processed foods, the consumption of unprocessed or minimally processed foods is associated with favorable patterns of protein intake, diet quality and lower cardiometabolic risk in French adults (INCA3). Eur. J. Nutr..

[71] Satija A (2017). Healthful and unhealthful plant-based diets and the risk of coronary heart disease in U.S. adults. J. Am. Coll. Cardiol..

[72] Julia C (2018). Contribution of ultra-processed foods in the diet of adults from the French NutriNet-Santé study. Public Health Nutr..

[73] Crimarco A (2020). A randomized crossover trial on the effect of plant-based compared with animal-based meat on trimethylamine-N-oxide and cardiovascular disease risk factors in generally healthy adults: Study With Appetizing Plantfood—Meat Eating Alternative Trial (SWAP-MEAT). Am. J. Clin. Nutr..

[74] Gibney M, Allison D, Bier D, Dwyer J (2020). Uncertainty in human nutrition research. Nat. Food.

[75] He J, Liu H, Balamurugan S, Shao S (2021). Fatty acids and volatile flavor compounds in commercial plant-based burgers. J. Food Sci..

[76] Courtney-Martin G (2021). False equivalence or fake news: Is a peanut really an egg?. J. Nutr..

[77] Willett W (2019). Food in the Anthropocene: The EAT–Lancet Commission on healthy diets from sustainable food systems. Lancet.

[78] Roessner U, Wagner C, Kopka J, Trethewey RN, Willmitzer L (2000). Simultaneous analysis of metabolites in potato tuber by gas chromatography–mass spectrometry. Plant J..

[79] Fiehn O (2008). Quality control for plant metabolomics: Reporting MSI-compliant studies. Plant J..

[80] Kind T (2009). FiehnLib: Mass spectral and retention index libraries for metabolomics based on quadrupole and time-of-flight gas chromatography/mass spectrometry. Anal. Chem..

[81] McNulty NP (2011). The impact of a consortium of fermented milk strains on the gut microbiome of gnotobiotic mice and monozygotic twins. Sci. Transl. Med..

[82] Banerjee R (2015). Non-targeted metabolomics of Brg1/Brm double-mutant cardiomyocytes reveals a novel role for SWI/SNF complexes in metabolic homeostasis. Metabolomics.

[83] Clinton CM (2020). Non-targeted urinary metabolomics in pregnancy and associations with fetal growth restriction. Sci. Rep..

[84] Mallard, W. G. & Reed, J. *Automated Mass Spectral Deconvolution and Identification System: AMDIS User Guide.* iv + 58 pp (National Institute of Standards and Technology, US Department of Commerce, 1997).

[85] Halket JM (1999). Deconvolution gas chromatography/mass spectrometry of urinary organic acids–potential for pattern recognition and automated identification of metabolic disorders. Rapid Commun. Mass. Spectrom..

[86] Stein SE (1999). An integrated method for spectrum extraction and compound identification from gas chromatography/mass spectrometry data. J. Am. Soc. Mass Spectrom..

[87] Kopka J (2004). GMD@CSB.DB: The Golm metabolome database. Bioinformatics.

[88] Do KT (2018). Characterization of missing values in untargeted MS-based metabolomics data and evaluation of missing data handling strategies. Metabolomics.

[89] Wei R (2018). Missing value imputation approach for mass spectrometry-based metabolomics data. Sci. Rep..

[90] Barupal DK, Fiehn O (2017). Chemical similarity enrichment analysis (ChemRICH) as alternative to biochemical pathway mapping for metabolomic datasets. Sci. Rep..

